# Clinicopathologic features of TDO2 overexpression in renal cell carcinoma

**DOI:** 10.1186/s12885-021-08477-1

**Published:** 2021-06-26

**Authors:** Quoc Thang Pham, Daiki Taniyama, Yohei Sekino, Shintaro Akabane, Takashi Babasaki, Go Kobayashi, Naoya Sakamoto, Kazuhiro Sentani, Naohide Oue, Wataru Yasui

**Affiliations:** 1 Department of Molecular Pathology, Hiroshima University Graduate School of Biomedical and Health Sciences, 1-2-3 Kasumi, Minami-ku, Hiroshima, 734-8551 Japan; 2 Department of Pathology, University of Medicine and Pharmacy at Ho Chi Minh City, Ho Chi Minh City, Viet Nam; 3 Department of Urology, Hiroshima University Graduate School of Biomedical and Health Sciences, Hiroshima, Japan

**Keywords:** Cancer stem cell, PD-L1, PTEN, Renal cell carcinoma, TDO2

## Abstract

**Background:**

Tryptophan 2,3-dioxygenase (TDO2) is the primary enzyme catabolizing tryptophan. Several lines of evidence revealed that overexpression of TDO2 is involved in anoikis resistance, spheroid formation, proliferation, and invasion and correlates with poor prognosis in some cancers. The aim of this research was to uncover the expression and biofunction of TDO2 in renal cell carcinoma (RCC).

**Methods:**

To show the expression of TDO2 in RCC, we performed qRT-PCR and immunohistochemistry in integration with TCGA data analysis. The interaction of TDO2 with PD-L1, CD44, PTEN, and TDO2 expression was evaluated. We explored proliferation, colony formation, and invasion in RCC cells line affected by knockdown of TDO2.

**Results:**

RNA-Seq and immunohistochemical analysis showed that TDO2 expression was upregulated in RCC tissues and was associated with advanced disease and poor survival of RCC patients. Furthermore, TDO2 was co-expressed with PD-L1 and CD44. In silico analysis and in vitro knockout of PTEN in RCC cell lines revealed the ability of PTEN to regulate the expression of TDO2. Knockdown of TDO2 suppressed the proliferation and invasion of RCC cells.

**Conclusion:**

Our results suggest that TDO2 might have an important role in disease progression and could be a promising marker for targeted therapy in RCC.

(199 words)

**Supplementary Information:**

The online version contains supplementary material available at 10.1186/s12885-021-08477-1.

## Background

Kidney cancer has been newly diagnosed in more than 400,000 people and caused nearly 179,000 deaths from cancer worldwide in 2020 [1]. Approximately 90% of kidney cancer is renal cell carcinoma (RCC), which presents three main histological subtypes: clear cell RCC (75%), papillary RCC (10%), and chromophobe RCC (5%) [2]. The patients with RCC have an overall 5-year survival rate of around 75%, but it significantly decreases to 8% in those patients with metastasis [3].

Comprehensive genomic analysis in patients with RCC by The Cancer Genome Atlas (TCGA) teams revealed extraordinary molecular features of the three main subtypes [4–6]. Clear cell RCC shows high expression of ribose metabolism pathway mRNA and an increasing immune infiltrate signature, whereas type 1 papillary RCC shows an increase in the mRNA signature for RNA splicing and type 2 papillary RCC shows an increase in the expression of glycolysis and Krebs cycle genes [7]. In general, alteration of CDKN2A, increasing DNA hypermethylation, and an increasing immune infiltrate signature were associated with poor survival of patients with RCC [7].

Tryptophan 2,3-dioxygenase (TDO2) is the primary enzyme that catabolizes tryptophan into kynurenine located in the liver and brain [8]. TDO2 stimulates tumor development through kynurenine-aryl hydrocarbon receptor (AhR) axis in glioblastoma [9]. Tumors overexpressing TDO2 are resistant to tumor rejection, and treatment with a TDO2 inhibitor reversed the ability of tumor rejection in immunized mice [10]. Overexpression of TDO2 is involved in anoikis resistance, spheroid formation, proliferation, and invasion and is associated with poor prognosis in a variety of cancers such as breast, esophagus, colon, and rectal cancer [11–14]. A previous study showed that TDO2 expression was not upregulated in both RCC tissues and cell lines at mRNA levels [15]. In contrast, recent research combined with large-scale transcriptome analysis and a genome-scale metabolic network model revealed that the *TDO2* gene was evaluable in all three major RCC subtypes [16]. Therefore, it is necessary to uncover the expression and biofunction of TDO2 in RCC.

The present research is, to our knowledge, the first study to analyze the expression at both transcription and protein levels and determine the biofunction of TDO2 in RCC. To investigate TDO2 expression in RCC, we performed qRT-PCR and immunohistochemistry in combination with TCGA data analysis. Furthermore, we performed in vitro knockdown of TDO2 expression by small interfering RNA (siRNA) to analyze TDO2 biofunction in RCC cell lines.

## Materials and methods

### Tissue samples and cell lines

In a retrospective study design, primary tumors were collected from 98 RCC patients at Hiroshima University Hospital from 2002 to 2012 (Hiroshima, Japan). The RCC patients with preoperative chemotherapy were excluded from this study. Postoperative follow-up was scheduled every 1, 2, or 3 months during the first 2 years after surgery and every 6 months thereafter unless more frequent follow-up was deemed necessary. The median follow-up period was 52 months (range 1–119). Informed consent was obtained from all patients. This study was approved by the Ethical Committee for Human Genome Research of Hiroshima University, Hiroshima, Japan (No. IRINHI66). The 7th edition of the TNM classification system was used to determine tumor staging [17].

For quantitative reverse transcription polymerase chain reaction (qRT-PCR), 12 pairs of tumor tissue and normal tissue specimens, which were immediately frozen in liquid nitrogen and stored at − 80 °C after surgical removal, were used.

For immunohistochemistry, formalin-fixed, paraffin-embedded tissues from 86 RCC patients were used. Two tumor blocks in each patient, including the tumor and the tumor with non-neoplastic epithelial tissue, were evaluated by immunohistochemical staining.

Three RCC cell lines, including 786-O, ACHN, Caki-1 were used for the in vitro experiments. Cells were maintenance cultured in RPMI-1640 (Nissui Pharmaceutical Co., Ltd., Tokyo, Japan) plus 10% fetal bovine serum (FBS) (BioWhittaker, Walkersville, MD, USA) in a humidified incubator at 37 °C with 5% CO_2_. All cell lines were purchased from the Japanese Collection of Research Bioresources Cell Bank (Osaka, Japan).

### TCGA databases analysis

The RNA-Seq expression of target genes and clinicopathologic data of three cohorts, clear cell RCC (KIRC), papillary RCC (KIRP), and chromophobe RCC (KIRH), were downloaded from http://firebrowse.org/. The correlation between TDO2 and PDL1 gene expression and the mutation status of the top ten genes in clear cell RCC was explored with TIMER2.0, http://timer.comp-genomics.org/. The top ten mutated genes in clear cell RCC were retrieved from the NCI genome database (https://portal.gdc.cancer.gov/).

### qRT-PCR analysis

Total RNA was isolated using ISOGEN (Nippon Gene, Toyama, Japan), and 1 μg RNA was used to synthesize cDNA with a PrimeScript™ 1st strand cDNA Synthesis Kit (Takara Bio, Shiga, Japan). PCR was performed with a CFX Connect real-time PCR detection system (Bio-Rad) using the SYBR Green PCR Core Reagents Kit (Applied Biosystems; Thermo Fisher Scientific, USA). The 2^-ΔCT^ method was used to calculate the relative expression levels as previously described [12]. ACTB served as an internal control. Primer sequences for TDO2 were forward, 5′-CGGTGGTTCCTCAGGCTATC-3′ and reverse, 5′-CTTCGGTATCCAGTGTCGGG-3′. Primer sequences for ACTB were forward, 5′-CTGTCTGGCGGCACCACCAT-3′ and reverse, 5′-GCAACTAAGTCATAGTCCGC-3′.

### Immunohistochemical analysis

Sections of 3 μm thickness were used for immunohistochemical analysis. The immunohistochemical staining procedures were performed as previously described [12]. The expression of TDO2 was scored as described in the previous study, which combined the intensity (1+, 2+, 3+) and the percentage (from 0 to 100%) of tumor cells expressing TDO2 [12]. Immunohistochemical staining of ALDH1, CD44, CD133, EGFR, HER2, p53, and PDL1 was performed as previously described [18]. Primary antibodies and dilution ratios were described in detail in Table S1. The sections were incubated with primary antibody for 1 h at room temperature, followed by incubation with Dako Envision+ Peroxidase Detection System (Dako Cytomation, Carpinteria, CA, USA) (Anti-mouse or Anti-rabbit) 1 h at room temperature. The sections were incubated with the DAB Substrate-Chromogen Solution (Dako Cytomation) for 10 min for the color reaction. Two surgical pathologists (D.T. and K.S.) independently examined the immunohistochemical results without knowledge of clinicopathologic information or patient outcome.

### Western blot analysis

Cell pellets were lysed in RIPA buffer (50 mM Tris, pH 7.4, 125 mM NaCl, 0.1% NP40, 5 mM EDTA and protease inhibitor cocktail [cOmplete™, Roche]). Western blot procedures were carried out as previously described [19]. The primary and secondary antibodies are listed in Table S1. Immunocomplexes were detected with an ECL Western Blot Detection System (Amersham Biosciences, Little Chalfont, Buckinghamshire, UK). GAPDH was presented as an internal control.

### RNA interference

Short interfering RNA (siRNA) oligonucleotides targeting *TDO2* and a negative control were purchased from Invitrogen (Carlsbad, CA, USA). We used two different *TDO2* siRNA oligonucleotide sequences: siRNA1: 5′-AUACCUUGUACCUAUCACUCACAGU-3′ and siRNA2: 5′-CCCGACACUGGAUACCGAAGAUGAA-3′. Transfection was performed using Lipofectamine RNAiMAX (Invitrogen) following the manufacturer’s protocol.

### CRISPR-Cas9

To generate PTEN knockout ACHN and Caki-1 cells, we used All-in-One lentivector pLenti-U6-sgRNA-SFFVCas9-2A-Puro CRISPR/Cas9 (Cat. 380,721,110,703, ABM Inc., Richmond, BC, Canada) as previously described [20]. The PTEN-sgRNA sequence was TGGGAATAGTTACTCCC. PTEN knockout ACHN and Caki-1 cells were selected and maintenance cultured in RPMI plus 10% FBS containing 2 μg/mL puromycin.

### Proliferation, colony formation, and invasion assays

To examine the cell growth, 3000 cells were plated per well in 96-well plates. Cell growth was checked after 1, 2, and 4 days by 3-(4,5-dimethylthiazol-2-yl)-2,5-diphenyltetrazolium bromide (MTT) assays.

To generate colonies, 500 cells per well were seeded in 6-well plates with 2 mL RPMI 1640 plus 10% FBS. The plates were incubated at 37 °C in 5% CO_2_. The media was changed every 2–3 days. The numbers of colonies were counted after 14 days.

In vitro invasion assays were performed as previously described [21]. Cells were seeded at 10,000 cells in RPMI 1640 without FBS in the upper chamber of a culture insert (8-μm pore size; Corning, NY, USA) covered with 50 μL Matrigel (1 μg/mL). The bottom of the culture insert was embedded in RPMI 1640 with 10% FBS. After 24 h, the invaded cells on the lower surface of the insert were stained with CyQuant GR dye to determine the number of cells. Three independent experiments were carried out. The mean and SE were calculated for each of the experiments.

### Statistical methods

Associations between clinicopathologic parameters and TDO2 expression in TCGA datasets were examined by Wilcox/Kruskal-Wallis test. Receiver operating characteristic (ROC) curve analysis was used to determine the cut-off value for the TDO2 expression score that correlated with clinicopathologic features. Correlations between TDO2 staining and clinicopathologic features and/or and various molecules were checked by Chi-square test. The Kaplan-Meier method was performed to examine the overall survival of patients with high and low TDO2 expression. The differences in the intergroup comparisons were tested by Student *t*-test. A *p* value of < 0.05 was considered to indicate statistical significance. Statistical analyses were performed using SPSS version 20.0 (Chicago, IL, USA).

## Results

### TDO2 expression was associated with progression and poor survival

**Fig. 1:**
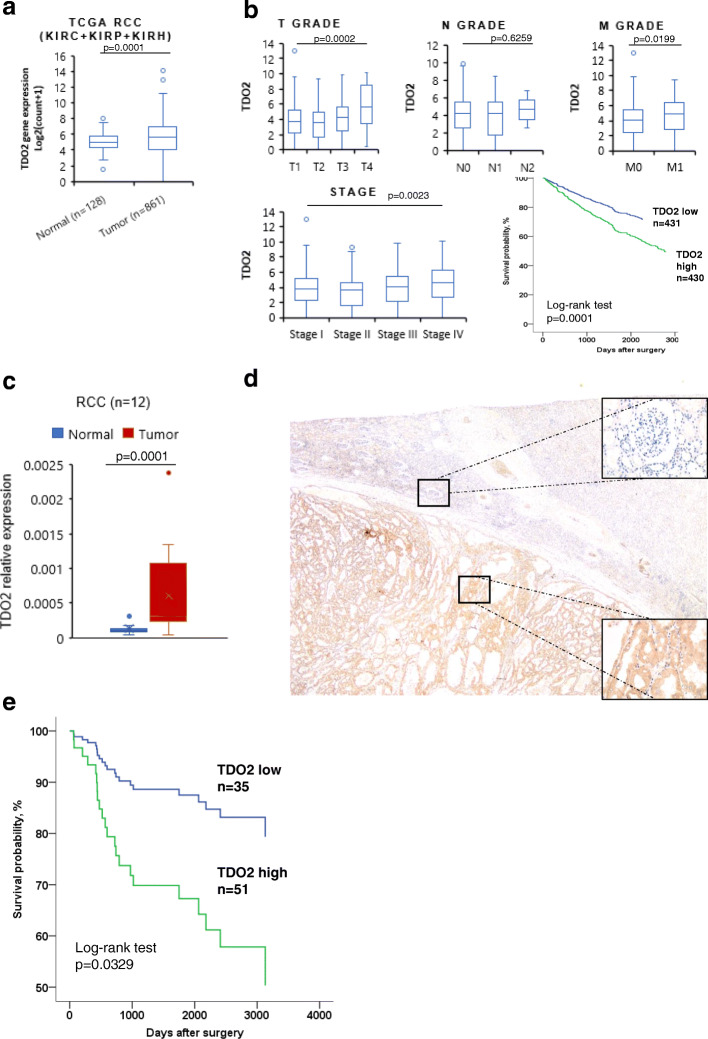
TDO2 expression and association with progression and prognosis. **a**, **b** TDO2 expression in TCGA RCC. **a** TDO2 expression in normal and tumor tissues. **b** TDO2 expression associated with clinicopathologic features; Kaplan-Meier analysis of RCC patients with high and low TDO2 expression. Unit: log2(count+ 1), Wilcox/Kruskal-Wallis test. **c** Validating TDO2 expression in 12 pairs of normal and tumor tissue samples by qRT-PCR. The data are displayed as mean ± SD (*n* = 3). **d** Immunohistochemical staining of TDO2 in a RCC sample, original magnification × 40. Normal kidney showed weak cytoplasmic staining (upper right corner, original magnification × 400), whereas tumor cells showed strong cytoplasmic staining with TDO2 (lower right corner, original magnification × 400). (e) Survival analysis of RCC patients with high and low TDO2 expression by Kaplan-Meier method

To compare the expression level of TDO2 in normal and tumor tissue, we retrieved the RNA-Seq data from TCGA RCC included in three databases: KIRC, KIRP, and KIRH. TDO2 expression was significantly present in RCC tissue (*p* = 0.0001) (Fig. 1a). In combination with clinicopathologic features, TDO2 expression was associated with advanced T grade (*p* = 0.0002), M grade (*p* = 0.0199), advanced stage (*p* = 0.0023), and poor survival in TCGA RCC data (Fig. 1b).

We next validated the expression of TDO2 by qRT-PCR and immunohistochemical analysis. qRT-PCR results from 12 pairs of normal and tumor tissue, whose clinicopathologic features are presented in Table S2, confirmed that TDO2 was clearly upregulated in RCC (*p* = 0.0001) (Fig. 1c). Immunohistochemical staining also revealed higher cytoplasmic expression of TDO2 in the tumor area compared with adjacent normal kidney (Figs. 1d and S1a). The staining of TDO2 was scored as described previously [12]. Then, we conducted ROC analysis to determine the cut-off value of the TDO2 expression score to investigate the correlation between TDO2 and other clinicopathologic features (Fig. S1b-d). The cut-off score was 75. As shown in Table 1, TDO2 expression also correlated with advanced T (*p* = 0.0004), M (*p* = 0.0308) grade, and stage (*p* = 0.0002) and higher histological grade (*p* = 0.0005).

**Table 1:** Correlation between TDO2 expression and clinicopathologic features in renal cell carcinoma

	TDO2 expression, n (%)		
	High	Low	*p* value^a^
Age			
≤ 65	21 (51.22)	20 (48.78)	0.1453
> 65	30 (66.67)	15 (33.33)	
Sex			
Female	13 (54.17)	11 (45.83)	0.5464
Male	38 (61.29)	24 (38.71)	
Histological classification			
ccRCC	38 (60.32)	25 (39.68)	0.7511
Non-ccRCC	13 (56.52)	10 (43.48)	
T grade			
T1	23 (44.23)	29 (55.73)	0.0004
T2/3/4	28 (82.35)	6 (17.65)	
N grade			
N0	46 (57.50)	34 (42.50)	0.2141
N1/2	5 (83.33)	1 (16.77)	
M grade			
M0	37 (53.62)	32 (46.38)	0.0308
M1	14 (82.35)	3 (17.65)	
Stage			
Stage I	22 (56.86)	29 (43.14)	0.0002
Stage II/III/IV	29 (82.86)	6 (17.14)	
Histological grade			
Grade 1/2	27 (46.55)	31 (53.45)	0.0005
Grade 3/4	24 (85.71)	4 (14.29)	

^a^ Chi-square test

To investigate the relationship between TDO2 expression and prognosis of RCC patients, we performed a survival analysis. Importantly, Kaplan-Meier analysis results from TCGA RCC database and our cohort showed that RCC patients with high TDO2 expression have shorter survival than those with low TDO2 expression (Fig. 1b, e). We further performed a Cox regression analysis to elucidate the independent predictive role of TDO2 in RCC (HR, 3.052; 95% CI, 1.124–8.290; *p* = 0.029). However, TDO2 expression was not found to be an independent factor of prognosis in RCC (HR, 1.920; 95% CI, 0.628–5.871; *p* = 0.253).

### TDO2 expression correlated with the expression of PD-L1 and CD44

**Fig. 2:**
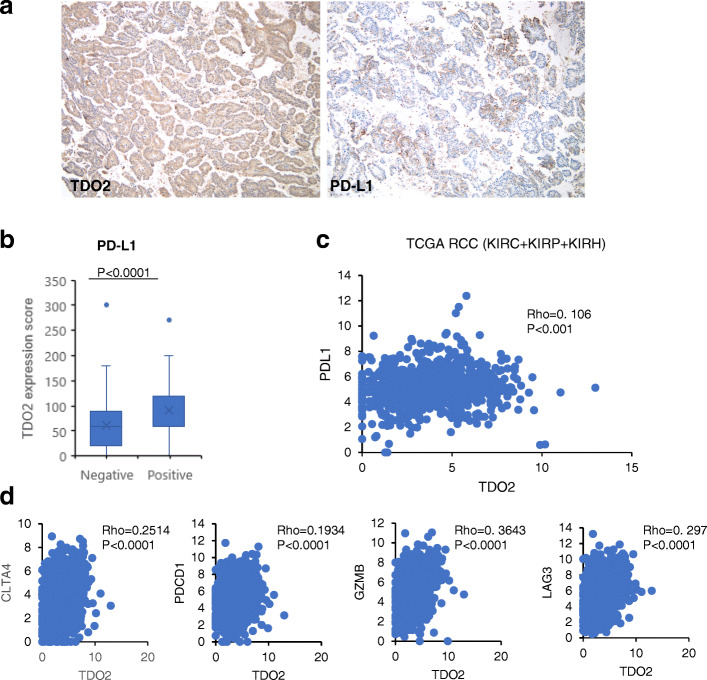
Correlation between TDO2 and PD-L1 expression. **a** Immunohistochemistry of TDO2 and PD-L1. Left picture, cytoplasmic staining of TDO2. Right picture, membranous staining of PD-L1. Original magnifications × 100. **b** Correlation between PD-L1 staining and TDO2 expression score. Wilcox test. **c**, **d** In silico analysis of the correlation between TDO2 and PD-L1 and T-cell exhaustion signature genes. **c** TDO2 expression correlated with PD-L1 expression. **d** TDO2 expression correlated with T-cell exhaustion signature genes (CLTA4, PDCD1, GZMB, LAG3). Unit: log2(count+ 1). Spearman rank correlation test

TDO2 overexpression was shown to suppress anti-tumor immune responses in mouse models [10], and PD-L1, an immune checkpoint, was shown to be significantly upregulated in clear cell RCC [16, 22]. Hence, we performed immunohistochemical staining with PD-L1 and found that PD-L1 expression correlated with the expression of TDO2 (Fig. 2a, b). We continued checking PD-L1 expression in the TCGA RCC data and other TCGA data sets by TIMER2.0, which revealed that TDO2 not only correlated with PD-L1 expression in RCC but also with other cancers (Fig. 2c, Fig. S2a). Interestingly, TDO2 expression was also associated with the expression of T-cell exhaustion markers such as CLTA4, PDCD1, GZMB, and LAG3 in RCC (Fig. 2d).

TDO2 expression has been shown to be associated with CD44 expression in esophageal cancer [12]. Therefore, we evaluated the correlation between the expression of TDO2 and several molecular markers presented in Table 2. Among three stem cell markers (ALDH1, CD44, and CD133) and other molecules (EGFR, HER2, and p53), TDO2 expression correlated only with the expression of CD44 (Fig. S2b, Table 2).

**Table 2:** Correlation between TDO2 expression and various molecules including cancer stem cell markers in renal cell carcinoma

	TDO2 expression, n (%)		
	High	Low	*p* value^a^
ALDH1			
Positive	32 (57.14)	24 (42.86)	0.5775
Negative	19 (63.33)	11 (36.67)	
CD44			
Positive	18 (78.26)	5 (21.74)	0.0306
Negative	33 (52.38)	30 (47.62)	
CD133			
Positive	10 (71.43)	4 (38.67)	0.3127
Negative	41 (56.94)	31 (43.06)	
EGFR			
Positive	10 (33.33)	20 (67.67)	0.3089
Negative	31 (55.36)	25 (44.64)	
HER2			
Positive	9 (50.00)	9 (50.00)	0.3663
Negative	42 (61.76)	26 (38.24)	
p53			
Positive	21 (67.74)	10 (32.26)	0.2317
Negative	30 (54.55)	25 (45.45)	

^a^ Chi-square test

### PTEN alteration correlated with the expression of TDO2

**Fig. 3:**
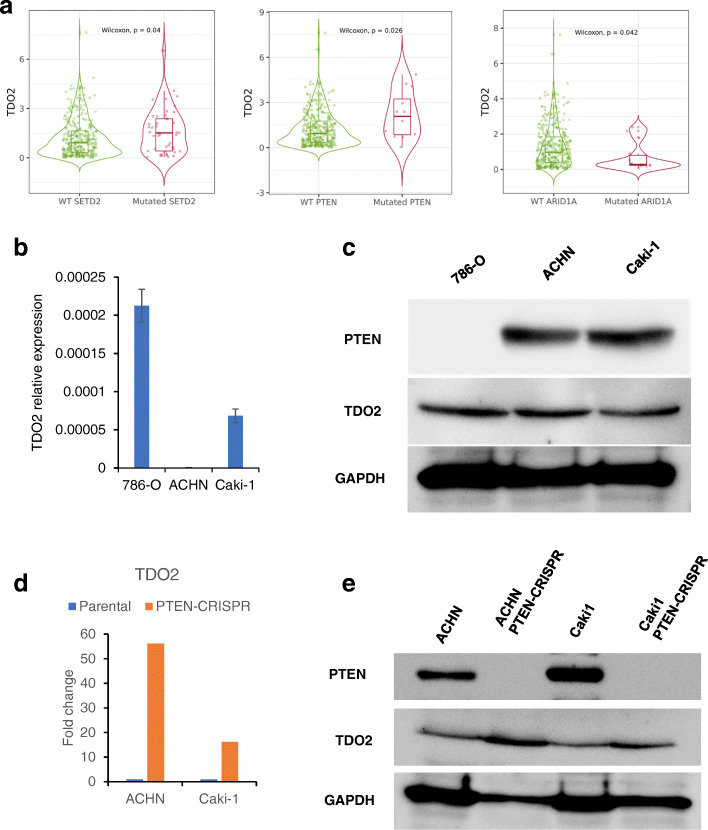
Correlation of TDO2 expression with PTEN alteration. **a** TDO2 expression correlated with mutation of STED2, PTEN and ARID1A genes by TIMER2.0. **b** qRT-PCR results of TDO2 expression in RCC cell lines. **c** Western blot analysis of the expression of PTEN and TDO2 in three RCC cell lines. GAPDH served as an internal control. The western blot bands were cropped and full-length blots/gels were presented in Fig. S5, Fig. S6. **d**, **e** The levels of TDO2 expression affected by PTEN knockout. **d** qRT-PCR results of TDO2 expression in PTEN knockout and its parental cells. **e** Western blot analysis of the expression of PTEN and TDO2 in PTEN knockout and its parental cells. GAPDH served as an internal control. The western blot bands were cropped and full-length blots/gels were presented in Fig. S7, Fig. S8. The bars indicate the mean ± SD (*n* = 3)

Previous research using TCGA data in RCC emphasized that patient prognosis was affected by some of the major genetic alterations in RCC [4–6]. We next retrieved the top ten mutated genes in clear cell RCC and evaluated the correlation of TDO2 expression with those genes by TIMER2.0 (Figs. S3a-b and 3a). PTEN and STED2 mutations were associated with upregulation of TDO2 expression whereas ARID1A mutation was associated with downregulation of TDO2 expression (Fig. 3a). Notably, only PTEN alteration correlated with decreased survival in clear cell RCC (Fig. S3c).

We investigated the expression of TDO2 in three RCC cell lines. Both qRT-PCR and Western blot analysis revealed that 786-O cells showed high expression of TDO2 (Fig. 3b, c). PTEN was expressed in ACHN and Caki-1 cells lines but not in 786-O, a PTEN mutated cell line (Fig. 3c). To clarify the relationship between PTEN and TDO2 expression, we used a PTEN-CripCas9 vector to knock out PTEN expression in ACHN and Caki-1 cells. As shown in Fig. 3d, PTEN expression was not detected in ACHN- and Caki-1-PTEN-CripCas9-transfected vectors. PTEN knockout cells showed upregulation of both protein and mRNA levels of TDO2 expression (Fig. 3d, e).

### Effect of knockdown of TDO2 expression on proliferation, colony formation, and invasion of RCC cells

**Fig. 4:**
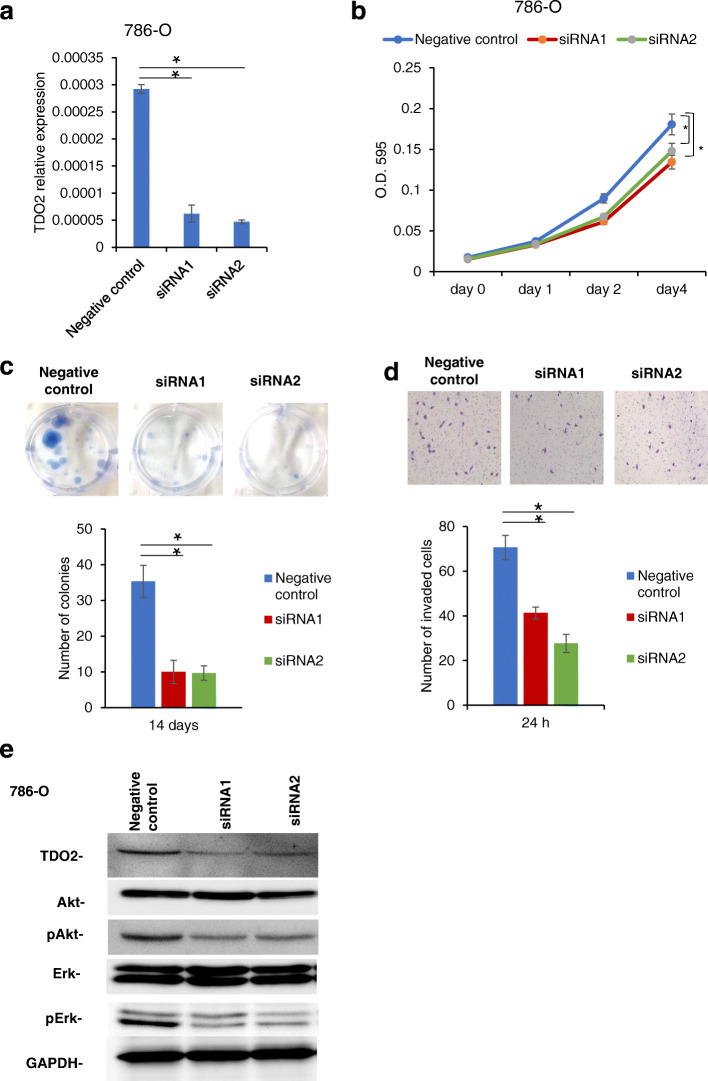
Effect of knockdown of TDO2 expression on proliferation, colony formation and invasion of RCC cells. **a** TDO2 mRNA levels in 786-O cells transfected with siRNA1, siRNA2, and siRNA negative control. The bars indicate the mean ± SD (*n* = 3). *p < 0.01. **b** MTT assay of 786-O cells transfected with siRNA1, siRNA2, and siRNA negative control. **c** Images of colony formation in 786-O cells transfected with siRNA1, siRNA2, and siRNA negative control at 14 days, and quantification of the average number of clones. The error bars indicate SE (*n* = 3). *p < 0.01. **d** Images of invasion assay in 786-O cells transfected with siRNA1, siRNA2, and siRNA negative control at 24 h, and quantification of the average number of invaded cells. The error bars indicate SE (*n* = 3). *p < 0.01. **e** Western blot analysis of the expression of TDO2, Akt, pAkt, Erk1/2, and pErk1/2 in 786-O cells transfected with siRNA1, siRNA2, and siRNA negative control. GAPDH served as an internal control. The western blot bands were cropped and full-length blots/gels were presented in Fig. S9, Fig. S10

To investigate the functional role of TDO2 in RCC, we performed knockdown of TDO2 expression in 786-O cells by using siRNA. As shown in Fig. 4a, the levels of TDO2 mRNA were significantly suppressed by siRNA1 (*p* = 0.0018) and siRNA2 (*p* = 0.0013). We next analyzed the effect of TDO2 knockdown on cell growth by MTT assays. The 786-O cells transfected with siRNA1 (*p* = 0.001) and siRNA2 (*p* < 0.0001) showed significantly decreased cell proliferation compared with the 786-O cells transfected with negative control siRNA (Fig. 4b). Furthermore, the colony formation ability of the 786-O cells with TDO2 knockdown was also clearly reduced compared with that in the 786-O cells transfected with negative control siRNA (Fig. 4c). We carried out an invasion assay to examine the potential role of TDO2 in invasiveness activities. After 24 h of seeding, the numbers of invaded 786-O cells transfected with siRNA1 (*p* = 0.0023) and siRNA2 (*p* = 0.0009) were significantly lower than those of 786-O cells transfected with negative control siRNA (Fig. 4d).

It is well known that activated EGFR pathways promote the proliferation and invasiveness activities of cancer cells [23]. We elaborated the effect of TDO2 knockdown on EGFR downstream molecules as described previously [12]. As shown in Fig. 4e, the levels of pErk and pAkt were significantly decreased in the786-O cells transfected with siRNA1 and siRNA2 compared to the control cells. We also examined the effect of TDO2 inhibition on sensitivity to tyrosine kinase inhibitor and Akt inhibitor. MTT assays were conducted to measure the cell viability of 786-O cells transfected with siRNA1 and siRNA2 and control cells under various concentrations of sunitinib and Akt inhibitor. However, the IC50 values of the 786-O cells transfected with siRNA1 and siRNA2 and that of the control cells were not significantly different (Fig. S4a-b).

## Discussion

In this work, we investigated the expression of TDO2 both at transcriptional and protein levels for the first time to resolve the controversy of TDO2 expression in previous studies [2, 3] in RCC. We revealed that TDO2 expression was upregulated in RCC tissues and correlated with tumor grade, metastatic status, stage, and histologic grade. TDO2 expression was persistently associated with poor survival of RCC patients across TCGA RCC databases and our cohort. Our data suggested that TDO2 immunostaining may be an applicable method for predicting the prognosis of a patient with RCC.

Approximately 20% of RCC patients without metastasis were reported to suffer recurrent disease within 5 years, and RCC patients with metastasis had the worst prognosis [3, 24]. Currently, the first-line options for treatment of advanced RCC patients are several tyrosine kinase inhibitors targeting the vascular endothelial growth factor (VEGF) pathway such as sunitinib and pazopanib [3]. Knowledge gained from large-scale genomic sequencing in RCC showed that the immune infiltrate signature was associated with poor survival and was a potential therapeutic target in RCC [7]. Furthermore, the combination of VEGF and PD-L1 inhibitors has resulted in the significant improvement in prognosis of patients with advanced RCC [25, 26]. Importantly, we found that TDO2 expression was associated with PD-L1 expression, T cell exhaustion markers, and enhanced proliferation and invasive activities of RCC cells. Indeed, the TDO2-kynurenines-AhR axis had an immune regulatory role of restricting the activation of T cells, and TDO2 inhibitor treatment showed recruitment of the anti-tumor effect in mouse models [10, 27]. Taken together, TDO2 may be a potential marker as a monotherapeutic target or in combination with a PD-L1 inhibitor to cure advanced RCC.

Cancer stem cells are known to contribute to the failure of conventional treatments [28]. We have already reported that TDO2 expression correlated with cancer stem cells in esophageal cancer [12]. In the present study, we also showed a correlation between the expression TDO2 and CD44, a cancer stem cell marker. Although TDO2 expression did not correlate with other cancer stem cell markers such as ALDH1 or CD133, our data encourage future study to fully determine the role of TDO2 in RCC stem cells.

PTEN has been shown to be a predictive factor and to have a crucial role in cell growth, survival, and drug resistance in cancer diseases through the PI3K/PTEN/AKT pathway [29]. Results from genomic sequencing data also point out that PTEN mutation is associated with disease progression and poor outcomes in clear cell and chromophobe RCC [4, 7]. Our results revealed an association between PTEN mutation status and TDO2 expression. We showed that knockout of PTEN upregulated the expression of TDO2 in RCC cells line, suggesting the ability of PTEN to regulate TDO2 expression. Kudo et al. [30] proved that TDO2 expression was regulated by the transcription factor C/EBPβ in glioblastoma. Several pieces of evidence were revealed that the PI3K/AKT pathway was also regulated by C/EBPα expression [31]. Collectively, our study may help uncover the mechanism of TDO2 regulation by PTEN in RCC.

The EGFR pathway is critical for the growth and invasive activities of cancer cells [23]. We showed that the phosphorylated levels of AKT and ERK were affected by knockdown of TDO2 expression in the RCC cell lines. AhR, activated by kynurenine-tryptophan metabolized product by TDO2, bypasses EGFR to retrieve PI3K/AKT and MEK/ERK pathway leading to resistance to tyrosine kinase inhibitors in non-small cell lung cancer [32]. We showed that in vitro inhibition of TDO2 clearly inhibited the cell growth, colony formation, and invasion of RCC cells. It was reported that phosphorylation of AKT and ERK controls apoptosis [33]. Interestingly, a recently developed TDO2 inhibitor has shown an ability to impair cell proliferation by inducing apoptosis and cell cycle arrest [34]. These data suggest that TDO2 may take part in the activation of EGFR and promote tumor growth in RCC.

Our study has several limitations. Our study may have potential bias due to patient selection and different in treatment regimens overtime based on retrospective study design. We need a prospective study to fulfill the predictive value of TDO2 in RCC. We have showed that knockout of PTEN upregulated the expression of TDO2 invitro. However, we have not clarified the effect of PTEN overexpression on the expression of TDO2 yet. In the future study, we will analyze the interaction between PTEN and TDO2 in RCC.

## Conclusion

In summary, the present work revealed that TDO2 expression was upregulated and associated with advanced disease and poor survival in RCC. The in vitro study revealed that knockout of PTEN enhanced TDO2 expression, and knockdown of TDO2 suppressed the proliferation and invasion of RCC cells. RNA-Seq and immunohistochemistry results showed that TDO2 expression correlated with PD-L1 expression. TDO2 could be a potential marker for targeted therapy in RCC.

## Supplementary Information

**Additional file 1.**

**Additional file 2.**

**Additional file 3.**

**Additional file 4.**

**Additional file 5.**

**Additional file 6.**

**Additional file 7.**

**Additional file 8.**

**Additional file 9.**

**Additional file 10.**

**Additional file 11.**

## Data Availability

All data generated or analysed during this study are included in this published article. The TCGA cohort (clear cell RCC (KIRC), papillary RCC (KIRP), and chromophobe RCC (KIRH)), publicly available data was downloaded for RNA-seq data and clinicopathologic data (Broad GDAC Firehose, http://firebrowse.org/). The mutation data of clear cell RCC was explored by GDC Exploration Tools (https://portal.gdc.cancer.gov/exploration). TIMER2.0 web server was used to check the correlation between TDO2 and PDL1 gene expression and the mutation status of the top ten genes in clear cell RCC (http://timer.comp-genomics.org/). Any other queries about the data used in this study should be directed to the corresponding author.

## Abbreviations

TDO2: Tryptophan 2,3-dioxygenase
RCC: Renal cell carcinoma
TCGA: The cancer genome atlas
AhR: Aryl hydrocarbon receptor
MTT: 3-(4,5-dimethylthiazol-2-yl)-2,5-diphenyltetrazolium bromide
ROC: Receiver operating characteristic
VEGF: Vascular endothelial growth factor
