# Supplementary material for: Clinicopathologic features of TDO2 overexpression in renal cell carcinoma
Source: BMC Cancer. 2021 Jun 26;21:737. doi: 10.1186/s12885-021-08477-1 (PMC8236178; doi:10.1186/s12885-021-08477-1)
Supplement: Supplementary file 1 — Additional file 1. [file 12885_2021_8477_MOESM1_ESM.docx]

**Clinicopathologic features of TDO2 overexpression in renal cell carcinoma**

Quoc Thang Pham^1,2^, Daiki Taniyama^1^, Yohei Sekino^3^, Shintaro Akabane^1^, Takashi Babasaki^1,3^, Go Kobayashi^1^, Naoya Sakamoto^1^, Kazuhiro Sentani^1^, Naohide Oue^1^, Wataru Yasui^1^

^1^Department of Molecular Pathology, Hiroshima University Graduate School of Biomedical and Health Sciences, Hiroshima, Japan

^2^Department of Pathology, University of Medicine and Pharmacy at Ho Chi Minh City, Viet Nam

^3^Department of Urology, Hiroshima University Graduate School of Biomedical and Health Sciences, Hiroshima, Japan

| **Supplementary Table 1**  List of primary antibodies | | |
| --- | --- | --- |
| Primary antibodies  for IHC | Lot No. (Company) | Dilution |
| ALDH1 | LS-B2497 (LifeSpan BioSciences) | 1:200 |
| CD44 | NBP2-44654 (Novus Biologicals) | 1:50 |
| CD133 | NB120-16518 (Novus Biologicals) | 1:50 |
| EGFR | 4267S (Cell Signaling Technology) | 1:50 |
| HER2 | 2242S (Cell Signaling Technology) | 1:50 |
| p53 | M7001 (Dako) | 1:50 |
| PD-L1 | 13684S (Cell Signaling Technology) | 1:50 |
| TDO2 | H00006999-B01P (Abnova) | 1:200 |
| Primary antibodies  for Western blot | Lot No. (company) |  |
| PTEN | 138G6 (Cell Signaling Technology) | 1:1000 |
| Akt | 9272 (Cell Signaling Technology) | 1:1000 |
| p-Akt | 9271 (Cell Signaling Technology) | 1:1000 |
| ERK | 9102 (Cell Signaling Technology) | 1:1000 |
| p-ERK | 9101 (Cell Signaling Technology) | 1:1000 |
| TDO2 | H00006999-B01P (Abnova) | 1:1000 |
| GAPDH | sc-365062 (Santa Cruz) | 1:20000 |
| Second antibodies  for Western blot | Lot No. (company) |  |
| Anti-IgG (H+L chain) (Mouse) pAb-HRP | 330 (MBL) | 1:1000 |
| Anti-IgG (H+L chain) (Rabit) pAb-HRP | 458 (MBL) | 1:1000 |

| **Supplementary Table 2**  Characteristics of 12 patients with clear cell renal cell carcinoma (ccRCC) | |
| --- | --- |
| Characteristic | N (%) |
| Histological classification |  |
| ccRCC | 9 (75) |
| Non-ccRCC | 3 (25) |
| T grade |  |
| T1 | 6 (50) |
| T2/3/4 | 6 (50) |
| N grade |  |
| N0 | 9 (75) |
| N1/2 | 3 (25) |
| M grade |  |
| M0 | 11 (91.7) |
| M1 | 1 (8.3) |
| Stage |  |
| Stage I | 2 (16.7) |
| Stage II/III/IV | 10 (83.3) |
| Histological grade |  |
| Grade 1/2 | 0 |
| Grade 3/4 | 12 (100) |
