# Supplementary figures and images for: Clinicopathologic features of TDO2 overexpression in renal cell carcinoma
Source: BMC Cancer. 2021 Jun 26;21:737. doi: 10.1186/s12885-021-08477-1 (PMC8236178; doi:10.1186/s12885-021-08477-1)

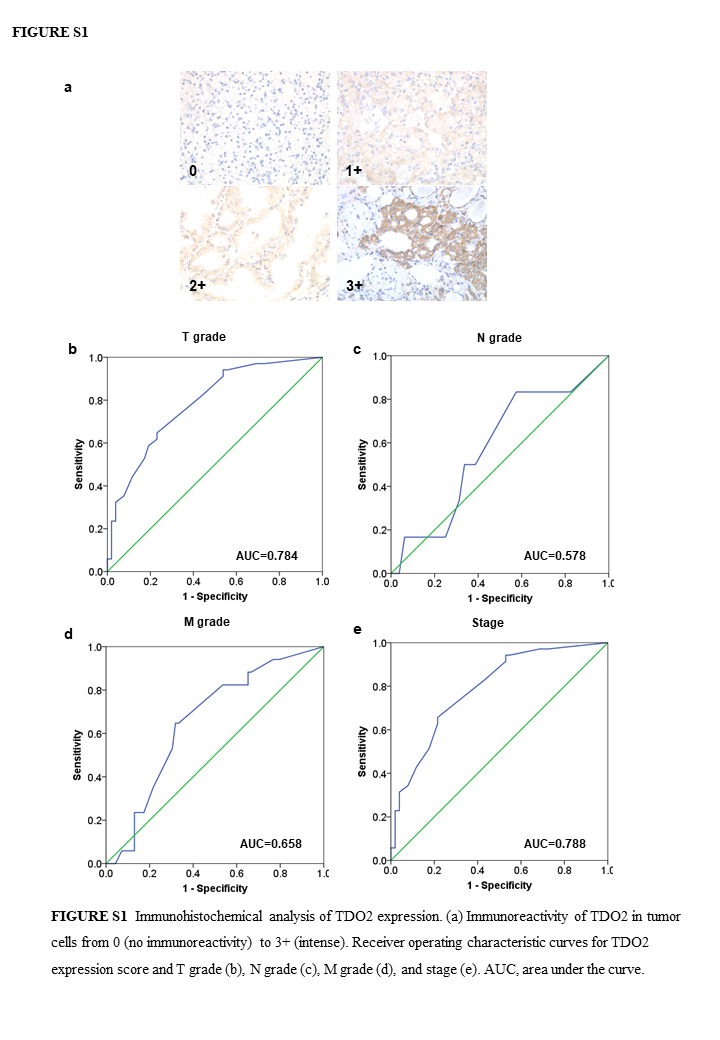

Supplement: Supplementary file 2 — Additional file 2. [file 12885_2021_8477_MOESM2_ESM.tif]

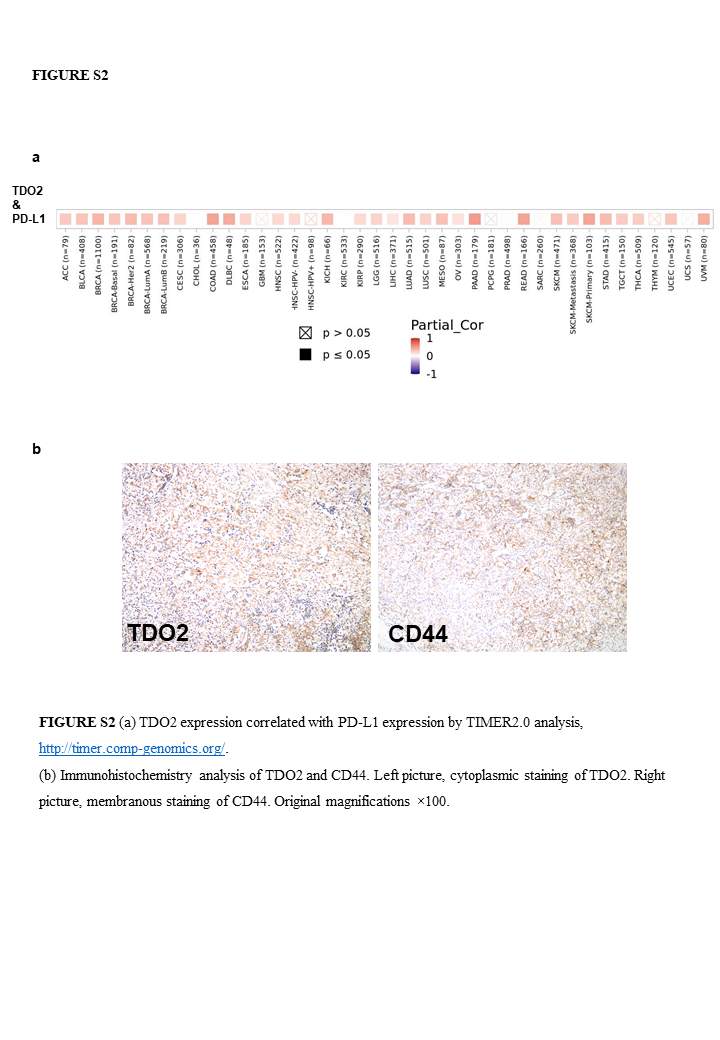

Supplement: Supplementary file 3 — Additional file 3. [file 12885_2021_8477_MOESM3_ESM.tif]

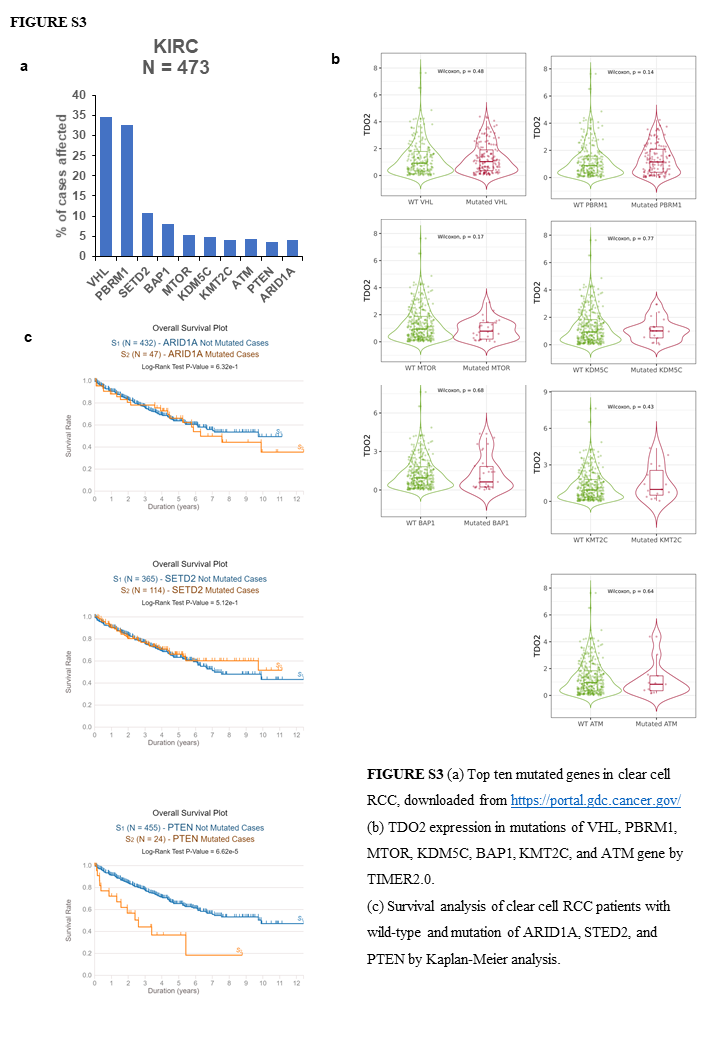

Supplement: Supplementary file 4 — Additional file 4. [file 12885_2021_8477_MOESM4_ESM.tif]

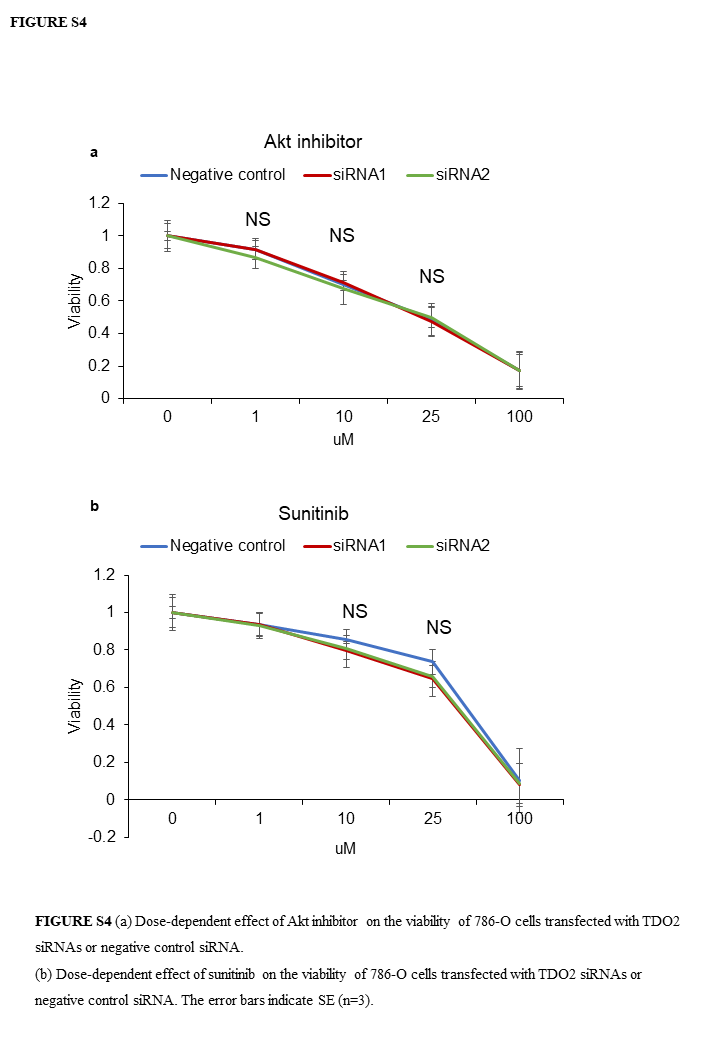

Supplement: Supplementary file 5 — Additional file 5. [file 12885_2021_8477_MOESM5_ESM.tif]

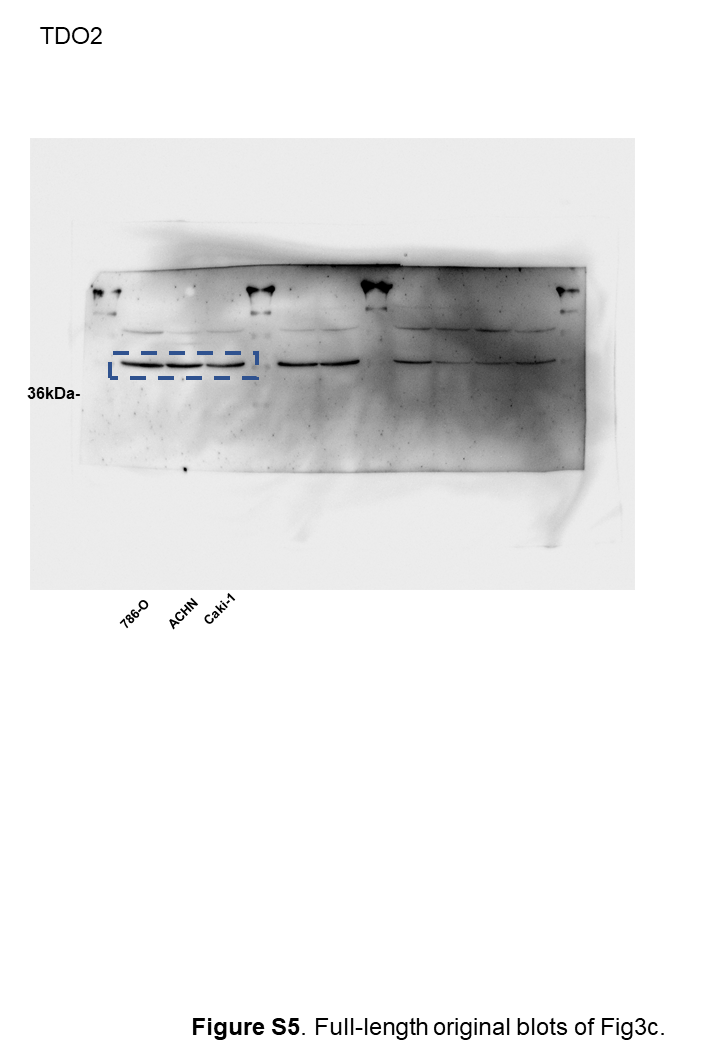

Supplement: Supplementary file 6 — Additional file 6. [file 12885_2021_8477_MOESM6_ESM.tif]

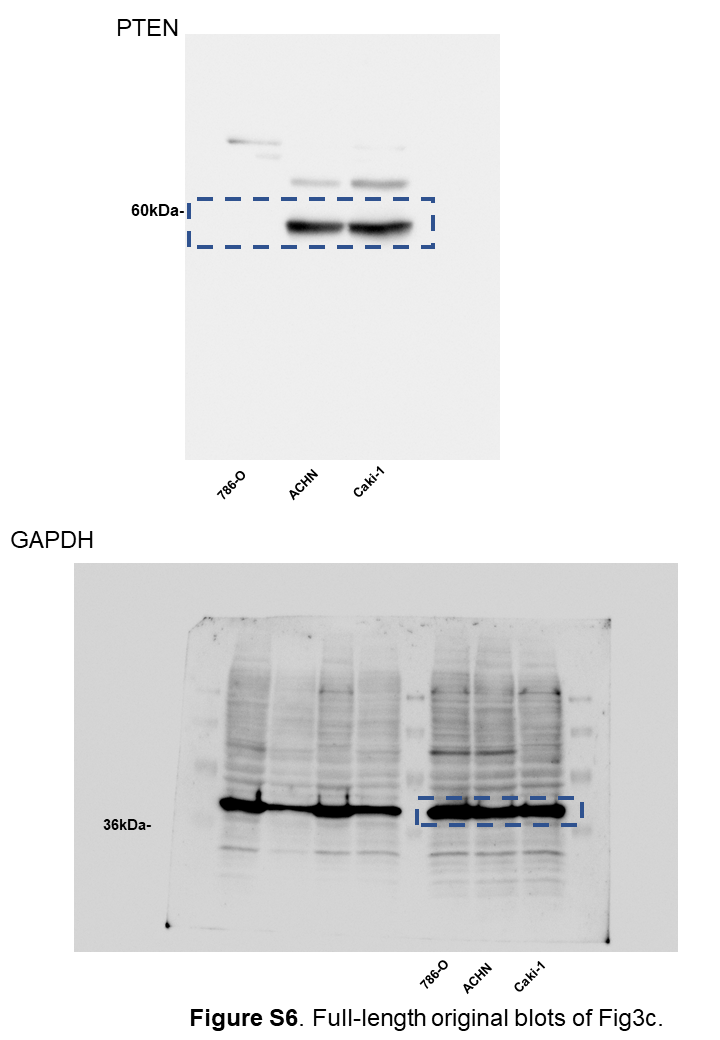

Supplement: Supplementary file 7 — Additional file 7. [file 12885_2021_8477_MOESM7_ESM.tif]

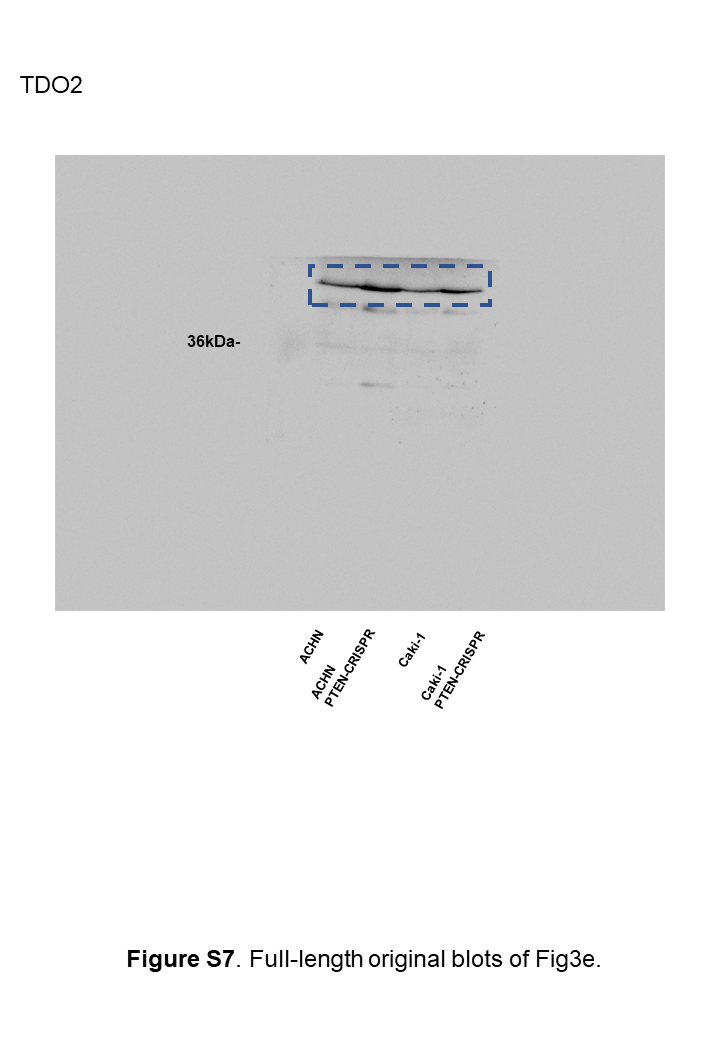

Supplement: Supplementary file 8 — Additional file 8. [file 12885_2021_8477_MOESM8_ESM.tif]

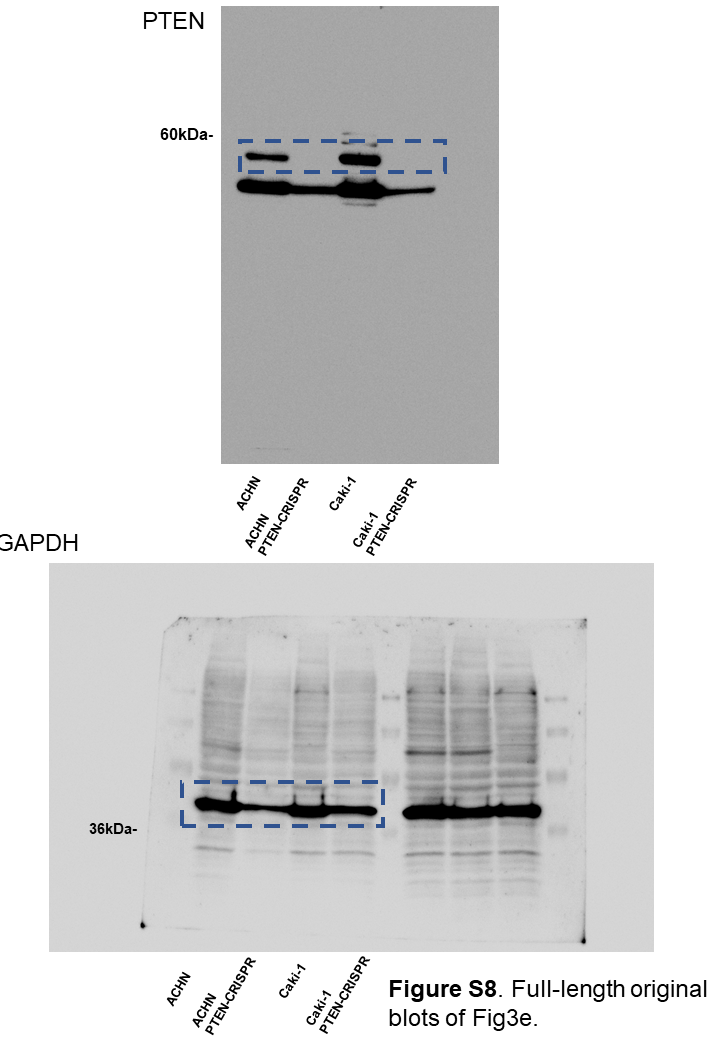

Supplement: Supplementary file 9 — Additional file 9. [file 12885_2021_8477_MOESM9_ESM.tif]

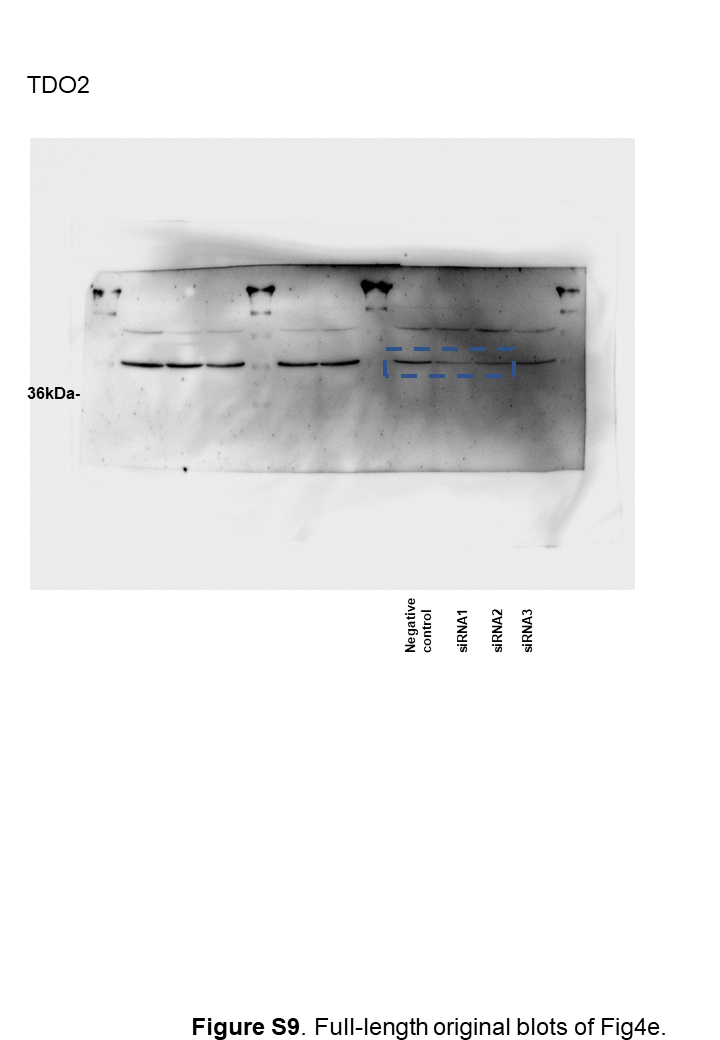

Supplement: Supplementary file 10 — Additional file 10. [file 12885_2021_8477_MOESM10_ESM.tif]

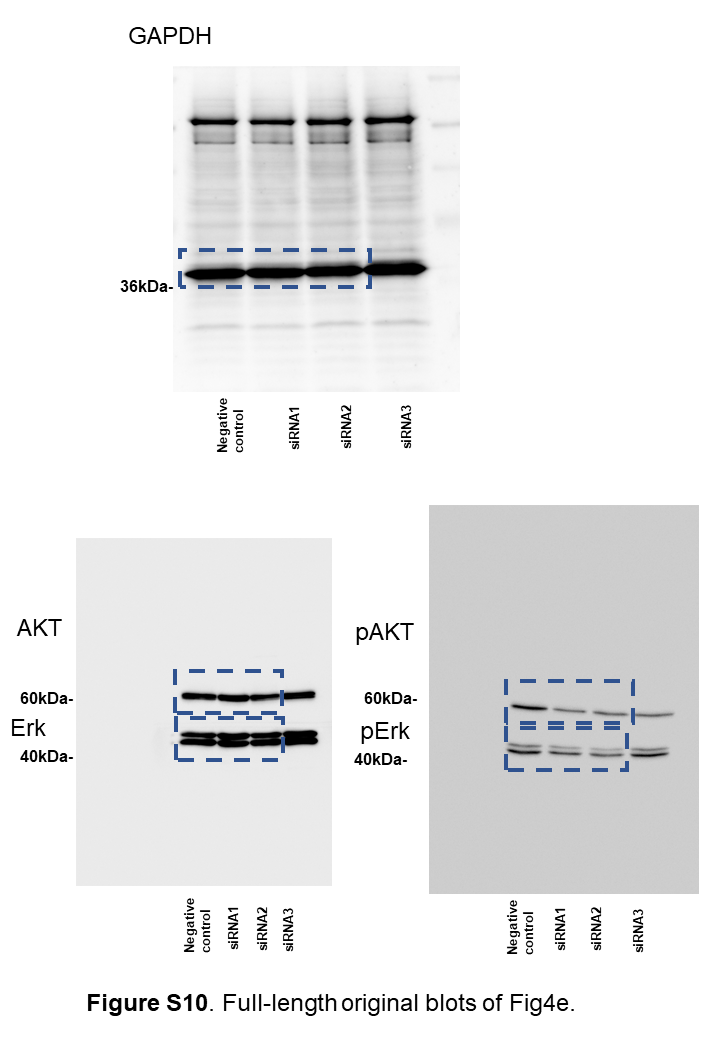

Supplement: Supplementary file 11 — Additional file 11. [file 12885_2021_8477_MOESM11_ESM.tif]
